# An Ether-Containing Hafnium–Diethylene Glycol Dry Resist Prepared by Molecular Layer Deposition for Mild-Acid Development

**DOI:** 10.3390/nano16120726

**Published:** 2026-06-11

**Authors:** Chao Shi, Yixian Wang, Zimai Wang, Yumo Tian, Kuanlin Chen, Linyang Li, Xianhaoyan Chen, Yuan Cai, Tuo Wang

**Affiliations:** 1 School of Chemical Engineering & Technology, Key Laboratory for Green Chemical Technology of Ministry of Education, Collaborative Innovation Center for Chemical Science & Engineering, Tianjin University, Tianjin 300072, China; 2 International Joint Laboratory of Low-Carbon Chemical Engineering of Ministry of Education, Tianjin 300350, China; 3 Zhejiang Institute of Tianjin University, Ningbo 315201, China; 4 Haihe Laboratory of Sustainable Chemical Transformations, Tianjin 300192, China; 5 Joint School of National University of Singapore and Tianjin University, International Campus of Tianjin University, Fuzhou 350207, China; 6 National Industry-Education Platform of Energy Storage, Tianjin 300350, China

**Keywords:** dry resist, molecular layer deposition, mild development, electron beam resist, extreme ultraviolet lithography

## Abstract

Advanced lithography, including electron beam lithography (EBL), X-ray lithography (XRL), and extreme ultraviolet lithography (EUVL), imposes stringent requirements on photoresists in resolution, sensitivity, and process compatibility, thereby driving the development of metal-containing hybrid resists and vapor-phase deposition strategies. However, existing Hf-based dry hybrid photoresists often struggle to provide sufficient dissolution contrast for clean pattern formation under mild development, as unexposed regions are not fully removed. In this work, an ether-containing hafnium-based photoresist was fabricated by molecular layer deposition (MLD). Incorporation of the diethylene glycol (DEG)-derived ether unit modifies the local coordination environment of the hybrid film and enhances the removability of the unexposed regions, enabling removal in 0.1 M HCl. FTIR and XPS analyses reveal that exposure disrupts Hf-O-C coordination motifs and converts the initial hybrid network into a more HfO*_x_*-rich, less soluble framework. This combination of enhanced solubility in the unexposed regions and exposure-induced stabilization in the exposed regions establishes sufficient dissolution contrast for mild-acid negative-tone development. E-beam tests show a critical dose of approximately 250 μC·cm^−2^ and reproducible patterning down to 50 nm. These results identify DEG-mediated ether incorporation as an effective route to improving developer compatibility in Hf-based MLD dry resists.

## 1. Introduction

Micro- and nanofabrication can be achieved through several complementary patterning strategies. Soft lithography provides a convenient and low-cost route to micro- and nanostructures using elastomeric stamps and replica molding [1], while nanoimprint lithography enables direct mechanical replication of nanoscale features [2]. Wet-lithographic and confinement-assisted self-organization methods have also contributed to the patterning of functional materials [3]. Although these approaches have played important roles in nanotechnology, radiation-based advanced lithography, including electron beam lithography (EBL), X-ray lithography (XRL), and extreme ultraviolet lithography (EUVL), remains central to high-resolution semiconductor patterning, where photoresists must simultaneously satisfy stringent requirements in resolution, sensitivity, line-edge roughness, etch resistance, and process compatibility [4,5,6,7,8].

Conventional organic photoresists, such as chemically amplified resists (CARs), have been widely adopted owing to their simple fabrication processes [9]. However, as pattern dimensions continue to shrink, organic resist systems face increasing challenges associated with resolution–line edge roughness–sensitivity trade-offs, stochastic effects, and pattern-transfer durability [10,11,12,13,14]. Metal-containing organic–inorganic hybrid resists have therefore emerged as promising alternatives. By incorporating high-Z metal centers or inorganic motifs into an organic matrix, these materials can enhance radiation absorption and secondary-electron generation while improving plasma-etch resistance during pattern transfer [15,16,17,18,19]. Nevertheless, many hybrid resists are still prepared by solution spin coating, where residual solvent, film swelling, and interfacial mass transport during development may affect nanoscale pattern fidelity [20,21,22,23,24]. These limitations have motivated increasing interest in dry-resist strategies based on vapor-phase film formation.

Among vapor-phase approaches, molecular layer deposition (MLD) is particularly attractive because it enables solvent-free, self-limiting, and thickness-controllable growth of organic–inorganic hybrid films through sequential reactions between gaseous precursors [25,26,27,28]. This molecular-level control makes MLD well-suited for constructing ultrathin dry resists with programmable composition and network structure. Previous studies have demonstrated the feasibility of MLD-derived hybrid films as electron-beam and EUV-responsive resists, including Al-, Hf-, Sn-, and Zn-containing systems [29,30,31,32,33]. These reports indicate that both the metal center and the initial hybrid network structure strongly influence exposure-induced chemistry, dissolution behavior, sensitivity, and pattern quality [34,35,36,37,38]. In particular, Hf-containing hybrid films are attractive because the high-Z Hf center can contribute to strong radiation absorption and favorable etch resistance, while exposure-induced conversion toward HfO*_x_*-rich structures can provide a basis for negative-tone development [33].

Despite these advances, development chemistry remains a critical limitation for MLD-derived and other metal-containing hybrid resists. The 2.38 wt% (0.26 M) tetramethylammonium hydroxide (TMAH) aqueous solution is widely used for positive-tone development, whereas many emerging metal oxide or hybrid resist systems require relatively strong acidic or alkaline developers to achieve sufficient dissolution contrast [39,40,41,42,43,44]. Harsh development conditions may narrow the process window [45,46], aggravate stochastic defect formation [47,48], promote dark erosion or roughness formation, and introduce additional integration and sustainability burdens [49,50]. Strong acids or bases induce dark erosion, which reduces contrast, thins unexposed residual films, and exacerbates stochastic roughness such as LER and line-width roughness (LWR), effects that are especially detrimental for ultrathin resists (often <30 nm) for advanced lithography, with limited etch margin [51]. From an integration standpoint, aggressive alkaline wet chemistries can raise compatibility concerns for exposed advanced-node materials [52,53,54]. Likewise, highly acidic wet-processing chemistries can attack TiN- or TaN-based gate or masking layers [55,56,57] and, during downstream wet cleaning or pattern-transfer steps, degrade the pore structure and porosity of porous low-k dielectrics [58,59]. In addition, their use imposes stringent material compatibility requirements on fluid-handling components in track systems, increasing the risk of corrosion and maintenance burden [60,61]. Meanwhile, the neurotoxicity of TMAH and the corrosiveness of strong acids increase handling risks [62], complicate wastewater denitrification processes, and raise operating costs. Therefore, developing highly sensitive resist systems that remain patternable under substantially milder developer conditions is an important yet insufficiently addressed challenge.

This paper presents the design and bottom-up fabrication of a novel hafnium-based MLD dry photoresist film, in which tetrakis(dimethylamido)hafnium(IV) (TDMAH) and diethylene glycol (DEG) are incorporated to construct a size-restricted covalent network architecture, thereby enabling development in 0.1 M HCl. Specifically, the reaction between TDMAH and DEG incorporates ether-containing linkages into the hybrid matrix. Owing to the flexible ether backbone and the spacing between the terminal hydroxyl groups, the Hf-DEG film forms a hybrid coordination network with improved responsiveness to mild-acid development. Mechanistic studies further indicate exposure-induced depletion of C-O-related motifs together with Hf-O network reorganization, driving the film toward a more HfO*_x_*-rich and less soluble framework that enables efficient pattern development. Under electron-beam exposure, the hafnium–diethylene glycol (Hf-DEG) resist exhibits a critical dose of approximately 250 μC·cm^−2^, maintains clear dissolution contrast in 0.1 M HCl, and achieves reproducible 50 nm line patterning. Compared with the 3 M HCl developer previously used for Hf-EG hafnicone, the present developer concentration is reduced by a factor of 30, corresponding to an approximately 96.7% decrease in HCl usage at the same developer volume. These findings demonstrate that DEG incorporation enables substantially milder acid development without compromising key lithographic metrics, including sensitivity and nanoscale patterning capability.

## 2. Materials and Methods

### 2.1. Chemicals

TDMAH (99.9999% purity) was purchased from Shanghai Oriphant Chemical Co., Ltd. (Shanghai, China). DEG (99% purity) was obtained from Shanghai Aladdin Biochemical Technology Co., Ltd. (Shanghai, China). P-type boron-doped silicon wafers were used as substrates and supplied by Hefei Kejing Material Technology Co., Ltd. (Hefei, China). The wafers were 0.5 mm thick, double-side-polished, and covered with a native oxide layer approximately 1.5–2 nm thick.

### 2.2. Film Deposition

Film deposition was carried out in a home-built MLD system. The deposition chamber temperature was maintained in the range of 60–120 °C. To prevent precursor condensation, the entire gas delivery line from the precursor containers to the chamber was uniformly heated. Argon was used as the carrier gas with a flow rate of 160 sccm, and the chamber pressure was stabilized at approximately 80 Pa using a rotary vane vacuum pump with a pumping speed of 8 L·s^−1^.

TDMAH and DEG were maintained at 70 °C and 60 °C, respectively, to ensure sufficient vapor pressure. The TDMAH/DEG MLD process consisted of two half-reactions: the metal precursor dose time ranged from 0 to 1 s, and the DEG dose time ranged from 0 to 2 s. Each half-reaction was followed by a 30 s purge step.

### 2.3. Electron-Beam Exposure and Development

Dose matrix patterns and large-area exposed samples (exposure area approximately 2 mm × 3 mm) were prepared by using a field-emission scanning electron microscope (Thermo Fisher Scientific, Waltham, MA, USA). Resolution test patterns were exposed using a ZEISS Sigma 500 system (Carl Zeiss Microscopy GmbH, Jena, Germany) equipped with the L-Edit Layout Editor. The electron-beam accelerating voltage was set to 20 kV, and the beam current was measured using a Faraday cup.

For dose matrix tests, a fixed magnification covering the entire display area was employed, with an electron-beam current of 1.6 nA; the exposure dose was controlled by adjusting the exposure time. Large-area samples were exposed under the same conditions. Resolution test patterns were fabricated on a ZEISS Sigma 500 system using layouts designed with the L-Edit Layout Editor, with an exposure current of 0.2 nA.

The dissolution behavior of the Hf-DEG resist was evaluated using different developers, including deionized water and HCl of different concentrations, and the lithographic results discussed below focus on 0.1 M HCl unless otherwise noted. After development, the samples were rinsed with deionized water for 30 s, followed by nitrogen blow-drying.

### 2.4. Characterization

Film thickness was measured using a J. A. Woollam M-2000DI spectroscopic ellipsometer (J. A. Woollam Co., Inc., Lincoln, NE, USA) at incidence angles of 60–70° with 5° increments. The spectral range was 193–1690 nm, and the data were fitted using the Tauc–Lorentz model. ATR-FTIR measurements were performed on a Nicolet iS50 Fourier-transform infrared spectrometer (Thermo Fisher Scientific, Waltham, MA, USA) equipped with a Polaris infrared source and a dedicated ATR DLaTGS detector. Background spectra were collected with 32 scans and sample spectra with 64 scans at a resolution of 4 cm^−1^.

Chemical composition and surface morphology were analyzed by X-ray photoelectron spectroscopy (XPS), scanning electron microscopy (SEM), and atomic force microscopy (AFM). XPS was conducted using a Thermo Scientific K-Alpha+ spectrometer (Thermo Fisher Scientific, Waltham, MA, USA) with a microfocused monochromatic Al Kα source. SEM imaging was carried out on a Hitachi SU8600 (Hitachi High-Tech Corporation, Tokyo, Japan) at an accelerating voltage of 3 kV. AFM measurements were conducted on a Bruker Dimension Icon system (Bruker Nano, Inc., Santa Barbara, CA, USA) in tapping mode over a scan area of 25 × 25 μm. AFM data were further processed by first-order plane leveling. The arithmetic mean roughness (Ra) and root-mean-square roughness (Rq) were calculated from selected flat regions on the retained resist surface and the cleared region, avoiding step edges and isolated spike-like artifacts.

## 3. Results

### 3.1. Growth Behavior of the Hf-DEG MLD System

**Figure 1:**
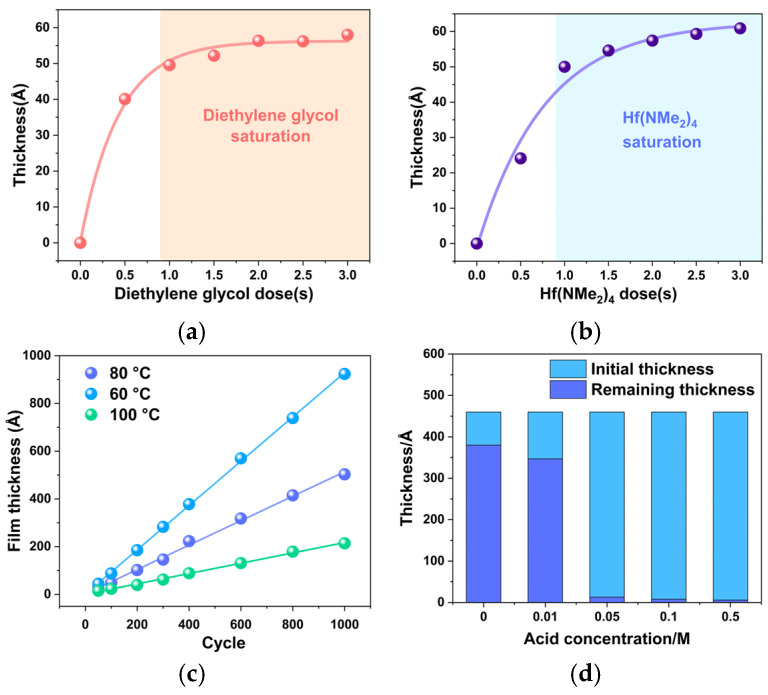
Deposition characteristics and dissolution performance of Hf-DEG films: (**a**,**b**) Influence of DEG and TDMAH dose time on the growth per cycle (GPC), respectively. Solid trend lines serve as visual guides to highlight the initial increase in GPC and its subsequent saturation with increasing precursor dose time. (**c**) Film thickness versus number of growth cycles at different deposition temperatures. (**d**) Dissolution performance of Hf-DEG films in HCl solutions of different concentrations.

To clarify how DEG incorporation influences both film growth and subsequent dissolution behavior, the effects of precursor pulse time, deposition temperature, and cycle number on film thickness, together with the dissolution behavior of the films in HCl solutions of different concentrations, were systematically investigated. Samples for precursor dose-time evaluation were deposited on silicon substrates at 80 °C for 100 cycles with different dose times (Figure 1a,b). For this system, the film growth rate increases with precursor dose time and gradually approaches saturation, consistent with the self-limiting nature of MLD reactions (Figure 1a,b). Notably, however, the growth remains sensitive to TDMAH dose duration even near saturation (Figure 1b), suggesting that the TDMAH half-cycle is not governed solely by the reaction of readily accessible surface sites. Rather, the continued increase in growth with prolonged TDMAH exposure implies that the precursor transport to less accessible reactive sites also contributes to the overall reaction, likely through diffusion along or within the growing hybrid network. The GPC of the Hf-DEG system decreases with increasing deposition temperature (Figure 1c). In addition, a larger GPC is observed at lower deposition temperatures (60 °C, Figure 1c), which is consistent with previous reports on hybrid MLD films [28,63]. Prior studies have attributed this accelerated growth behavior to the physisorption and diffusion of precursors within the film matrix [64,65,66]. In the Hf-DEG system, the increase in GPC can be similarly explained: DEG can undergo both interlayer and intralayer reactions, where intralayer reactions induce partial termination at certain reactive sites, leading to a more loosely packed and porous film structure. Beyond showing concentration-dependent film removal, this result demonstrates that DEG incorporation renders the as-deposited Hf-DEG network sufficiently responsive to acidic development that the unexposed film can be completely removed under relatively mild conditions (Figure 1d and Figure S1). This finding is significant because incomplete dissolution of unexposed regions is a central limitation of Hf-based dry hybrid resists, often restricting the dissolution contrast. The efficient removal achieved at low acid concentration therefore indicates that DEG effectively lowers the development threshold of the hybrid network, providing the chemical basis for mild-acid processing and supporting the use of 0.1 M HCl in the subsequent lithographic evaluation. It should be noted that even lower HCl concentrations, such as 0.05 M, can already promote substantial removal of the unexposed Hf-DEG film in the dissolution test. However, 0.1 M HCl was selected for the lithographic evaluation because it provided a more robust clearing margin and more reproducible pattern formation under the present development conditions. Further reduction in the acid concentration may be possible by increasing the development time or further tuning the initial network density, but this would need to be balanced against dark erosion, pattern roughness, and process reproducibility.

### 3.2. Exposure-Induced Chemical Transformation of Hf-DEG Resist

**Figure 2:**
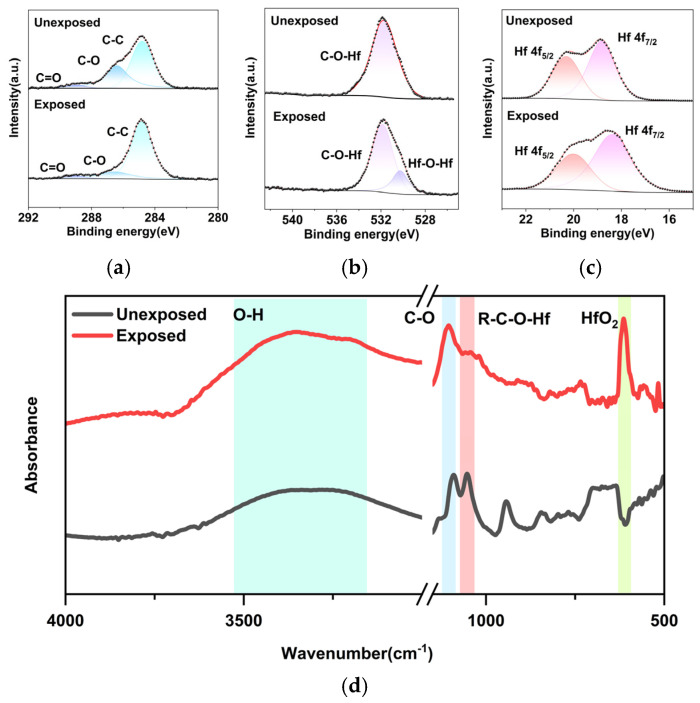
Exposure mechanism study of Hf-DEG dry resist. XPS results of (**a**) C 1s, (**b**) O 1s, and (**c**) Hf 4f peaks. (**d**) ATR-FTIR spectra of Hf-DEG samples before and after SEM e-beam exposure. All these results are based on samples exposed to an SEM electron beam at 1500 μC·cm^−2^ and developed in 0.1 M HCl for 90 s.

Electron-beam exposure is associated with pronounced chemical changes in the Hf-DEG hybrid resist film, as reflected by the attenuation of organic coordination features and the enhanced contribution of oxide-like Hf-O bonding. These spectral changes are associated with a transition of the initially hybrid organic–inorganic network toward a more HfO*_x_*-rich framework (Figure 2). This behavior, directly reflected in XPS and FTIR, is consistent with previous studies of hafnium-based hybrid resists, in which irradiation is associated with the loss of organic coordination motifs and progressive inorganic enrichment of the HfO*_x_* framework [33,67,68,69,70,71]. The XPS C 1s signal indicates substantial removal of the DEG-derived C-O contribution after exposure, consistent with the disruption of the organic coordination environment. In the unexposed film, the carbon environment is dominated by a main C-C/C-H contribution at ~284.8 eV together with a distinct DEG-derived C-O component at ~286.2 eV and a weak high-binding-energy shoulder at ~288.5–289.0 eV (Figure 2a), which could be assigned to oxidized carbon species [72,73]. After exposure, the C-O contribution is markedly reduced, the C-C/C-H component becomes the dominant feature in the C 1s envelope, and the oxidized-carbon contribution increases slightly. This evolution suggests a substantial attenuation of the DEG-derived organic coordination environment and is consistent with partial disruption or loss of linker-related C-O motifs in the exposed regions. Such an exposure-induced loss of organic coordination features is consistent with previous reports on hafnium-based hybrid resist systems [33,71].

The XPS O 1s spectra provide clearer evidence that oxygen coordination shifts from organic-associated environments toward oxide-like Hf-O bonding upon exposure [74] (Figure 2b). The unexposed film is characterized mainly by higher-binding-energy oxygen species associated with organic C-O and Hf-O-C environments, whereas the exposed film contains an additional lower-binding-energy component at ~529.8–530.5 eV together with a marked attenuation of the higher-binding-energy contributions. Peak fitting indicates that this newly emerged low-binding-energy component is consistent with oxide-like Hf–O bonding, suggesting an increased contribution of inorganic Hf-O environments after exposure. This trend may support, but does not by itself uniquely determine, a local reorganization from organic-associated Hf-O-C coordination toward a more inorganic Hf-O-rich network. This assignment is consistent with reported O 1s binding energies for HfO*_x_*-like environments in hafnium-based materials [70,74,75,76,77]. The corresponding atomic percentages and deconvoluted O 1s/C 1s components before and after e-beam exposure are provided (Tables S1–S3).

The Hf 4f spectra further suggest that exposure mainly alters the local coordination environment of Hf rather than its formal oxidation state (Figure 2c). In both exposed and unexposed films, the characteristic Hf 4f doublet remains clearly visible, indicating that Hf stays predominantly in the Hf(IV) state before and after irradiation. However, the exposed film exhibits a modest shift in peak position together with a change in line shape, consistent with a more inorganic-like coordination environment around the Hf centers. Combined with the O 1s evolution, this behavior indicates that exposure mainly reconstructs the local bonding configuration of Hf rather than changing its formal oxidation state, shifting the network from hybrid Hf-O-C coordination toward a more inorganic Hf-O-rich structure. Similar Hf 4f evolution has been reported during the conversion of hybrid hafnium-containing networks to oxide-like phases [70,74].

The FTIR spectra show the same overall conversion trend, corroborating the exposure-induced loss of organic linker features and growth of oxide-like Hf-O character (Figure 2d). In the unexposed film, the O-H stretching band in the 3200–3600 cm^−1^ region is weak and broad, indicating only a limited population of hydroxylated and/or hydrated species in the as-deposited network. After exposure, this band becomes substantially stronger and broader, indicating a more strongly hydrogen-bonded O-H environment in the reconstructed film. In the fingerprint region, the unexposed film exhibits pronounced C-O-related absorptions from the DEG-derived ligand framework, whereas these bands become much weaker and less defined after exposure. At the same time, a distinct absorption appears near ~700 cm^−1^ only in the exposed film, characteristic of Hf-O vibrations in hafnium oxide or HfO*_x_*-like environments. The emergence of this low-wavenumber Hf-O feature together with the attenuation of the C-O-related bands is consistent with FTIR trends reported for hybrid hafnium films undergoing conversion toward HfO*_x_*-like frameworks [33,69,70].

Taken together, the XPS and FTIR results establish the directly observed chemical changes after exposure: attenuation of DEG-related C-O/Hf-O-C features and enhancement of oxide-like Hf-O character (Figure 2). These changes are consistent with the formation of a more inorganic and less soluble HfO*_x_*-rich network in the exposed regions, which rationalizes the observed negative-tone development behavior. In the unexposed regions, a substantial fraction of the network is expected to remain linked through DEG-derived Hf-O-C motifs. These motifs may be associated with proton-assisted bond disruption and ligand exchange under acidic development conditions, although these acid-driven dissolution steps are inferred from the dissolution behavior rather than directly resolved by XPS or FTIR. The resulting contrast between the spectroscopically supported exposed-state stabilization and the inferred acid responsiveness of the unexposed network is consistent with the mechanism proposed for other hafnium-containing hybrid negative-tone resist systems [33,78], in which irradiation-induced ligand loss is accompanied by the formation of a less soluble HfO*_x_*-rich network; in the present Hf-DEG resist, the incorporation of DEG further enhances the acid responsiveness of the unexposed film, thereby enabling effective negative-tone development under comparatively mild acidic conditions.

**Scheme 1:**
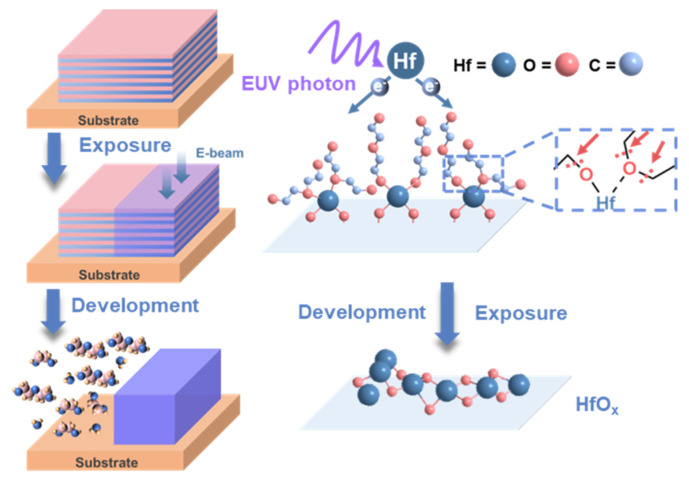
Schematic illustration of the proposed deposition, exposure, and development behavior of the Hf-DEG dry resist. Based on the XPS and FTIR results, electron-beam exposure is proposed to attenuate linker-related C-O/Hf-O-C motifs and promote a more HfO*_x_*-like coordination environment, leading to reduced solubility of the exposed regions during mild-acid development.

Based on these observed chemical changes, a schematic exposure-induced conversion mechanism is proposed (Scheme 1). Before exposure, the Hf-DEG film is represented as a hybrid coordination network in which Hf centers are connected through DEG-derived Hf-O-C motifs. After electron-beam irradiation, the attenuation of C-O-related XPS and FTIR signals suggests the loss or disruption of DEG-associated coordination environments, while the enhanced oxide-like Hf-O contribution is consistent with local reorganization toward a more inorganic Hf-O-rich framework. This conversion is interpreted mainly as a change in the local bonding environment of Hf rather than a change in its formal oxidation state. Oxygen re-coordination around Hf centers may accompany this process, but the exact molecular sequence cannot be uniquely determined from the present data. As a result, the exposed regions are expected to become less soluble and more chemically resistant under acidic development conditions. By contrast, the unexposed regions retain more DEG-derived Hf-O-C motifs, which are proposed to remain more acid-responsive. Proton-assisted bond disruption and ligand exchange are therefore suggested as plausible dissolution pathways for the unexposed film in 0.1 M HCl, but these steps should be regarded as mechanistic inferences rather than direct spectroscopic observations. Together, the spectroscopically supported exposure-induced stabilization and the inferred acid responsiveness of the unexposed network provide a plausible basis for the selective development of the Hf-DEG hybrid resist under mild acidic conditions.

### 3.3. Electron-Beam Exposure Study of Hf-DEG Hybrid Films

**Figure 3:**
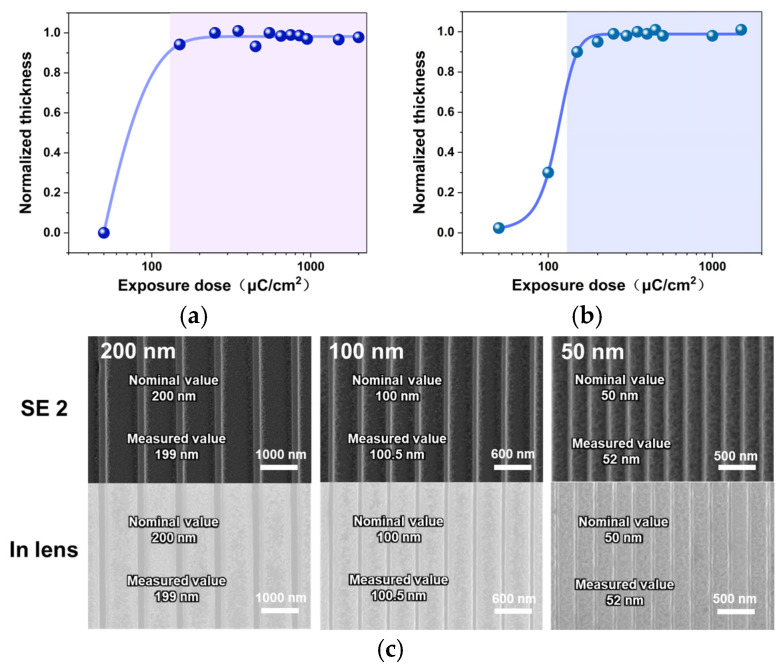
Exposure response and patterning performance of the Hf-DEG dry resist: (**a**,**b**) Normalized remaining thickness plotted against electron-beam dose for two independent dose-matrix experiments (dose increments: 50 and 100 μC·cm^−2^, respectively) after development in 0.1 M HCl. Solid lines serve as visual guides to emphasize the dose-dependent thickness increase and subsequent saturation; these are non-quantitative trends. (**c**) Representative SEM images of line patterns with nominal trench widths of 200, 100, and 50 nm, fabricated at a dose of 1500 μC·cm^−2^ and developed in 0.1 M HCl. Upper and lower rows correspond to SE2 and In-lens detector modes, respectively, providing complementary morphological perspectives.

Hf-DEG films retain a clear negative-tone lithographic response under mild-acid development, with a practical critical dose and reproducible thickness retention behavior. To evaluate this behavior, two independent dose-matrix experiments were performed using different dose increments of 50 and 100 μC·cm^−2^, respectively, to evaluate the repeatability of the retained-thickness response. Although these datasets were not point-by-point statistical replicates and therefore were not used to calculate error bars, both experiments show the same threshold-to-plateau behavior after development in 0.1 M HCl. The consistent dose-dependent increase in normalized remaining thickness and the reproducible retained-film plateau indicate that the negative-tone solubility transition of the Hf-DEG resist is repeatable under the present exposure and development conditions. Two independent dose-matrix experiments with different dose increments were performed over 50–1500 μC·cm^−2^, and both showed the same overall response trend (Figure 3a,b and Figure S2). After development in 0.1 M HCl, the results identify 250 μC·cm^−2^ as the critical dose of the Hf-DEG system under the present mild-acid development condition. The critical dose was defined as the lowest exposure dose at which the normalized remaining thickness reached a stable retained-film plateau after development. To place this dose response in the context of previously reported Hf-based MLD hybrid resists, Table 1 summarizes the developer concentration, tone, and critical dose of the present Hf-DEG system and a representative Hf-EG hafnicone resist. Compared with the previously reported Hf-EG system developed in 3 M HCl [33], the Hf-DEG resist operates in 0.1 M HCl while maintaining a lower critical dose and clear negative-tone behavior. This solubility transition is reproducible and remains compatible with the improved developer responsiveness introduced by DEG. The normalized remaining-thickness curves derived from the two independent dose-matrix experiments show consistent threshold-to-plateau behavior despite the different dose-step intervals (Figure 3a,b). DEG incorporation modifies the coordination environment and thereby increases the acid solubility of the unexposed film. Importantly, this increased solubility of the unexposed network is balanced by efficient exposure-induced stabilization. Upon exposure, the Hf-DEG system still exhibits a steep transition from soluble to insoluble behavior, as reflected by the rapid increase in retained pattern height within a narrow dose range. This transition is accompanied by the evolution of the Hf-DEG network toward hafnium oxide-like species, producing pronounced dissolution contrast while preserving the sensitivity required for lithographic pattern formation.

Patterning tests on nominal line/space structures with trench widths of 200, 100, and 50 nm showed continuous patterns down to 50 nm under the optimized development conditions (Figure 3c), whereas the smallest nominal 20 nm features were less reliably resolved and less continuous (Figure S3). Line/space structures obtained at other exposure doses were also evaluated (Figure S4). These results indicate that the Hf-DEG film can maintain sufficient pattern fidelity under mild-acid development while still reaching practically relevant nanoscale dimensions. Meanwhile, large-area statistical LER/LWR analysis is beyond the scope of this study. Such performance cannot be attributed solely to the exposure-induced formation of a less soluble HfO*_x_*-rich framework. Rather, the key feature of the Hf-DEG system is that DEG modifies the initial coordination environment of the unexposed film, leaving a larger fraction of acid-labile Hf-O-C motifs that remain susceptible to proton-assisted bond disruption and ligand exchange in dilute acid. Upon exposure, C-O bond cleavage is followed by Hf-O network reorganization toward a more chemically resistant HfO*_x_*-rich structure, as supported by the XPS and FTIR results (Figure 2). The combination of enhanced acid responsiveness in the unexposed regions and exposure-induced stabilization in the exposed regions therefore creates a sufficiently strong solubility contrast even in 0.1 M HCl, enabling mild development while preserving practical lithographic performance, including a critical dose of ~250 μC·cm^−2^ and 50 nm patterning capability. In this sense, the advance of this work lies not simply in another Hf-based MLD resist but in a DEG-engineered ether-containing Hf hybrid system that shifts developer compatibility to substantially milder acidic conditions.

### 3.4. The 3D Topography and Plasma-Etch Compatibility of Hf-DEG Patterns

**Figure 4:**
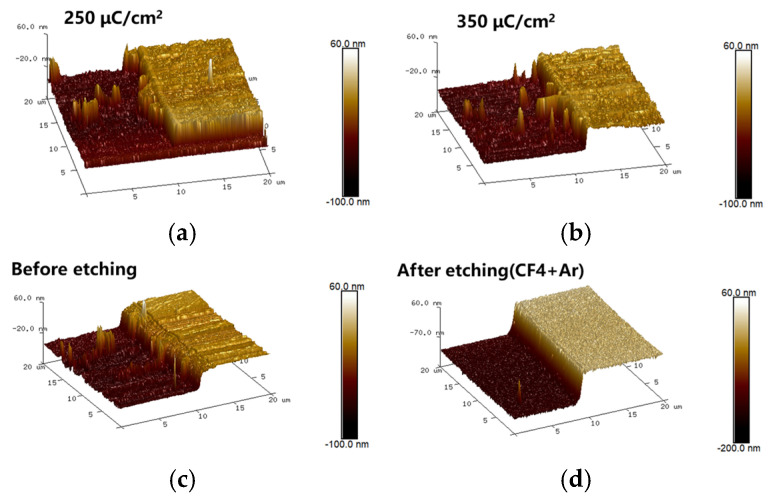
The 3D topography of Hf-DEG patterns: (**a**,**b**) Three-dimensional AFM topographies of step patterns exposed at 350 and 250 μC·cm^−2^, respectively, and developed in 0.1 M HCl. (**c**,**d**) Three-dimensional AFM topographies of a representative pattern before and after CF_4_/Ar plasma etching, respectively. The samples were etched for 1 min under CF_4_/Ar gas flows of 30/5 sccm and ICP/CCP powers of 400/200 W.

Patterns generated at the critical dose already exhibit sufficient topographic definition for stable negative-tone pattern retention (Figure 4a,b). To directly relate the dose-response behavior to the resulting morphology, representative structures exposed at 250 and 350 μC·cm^−2^ were examined by three-dimensional surface topography analysis (Figure 4a,b). A clear step-like height contrast between retained and exposed regions is already established at 250 μC·cm^−2^, confirming that this dose is sufficient to trigger the essential pattern-forming transition in the Hf-DEG resist. When the dose is increased to 350 μC·cm^−2^, the overall surface topography remains qualitatively similar, although the retained region becomes more laterally continuous and the cleared region shows fewer localized spike-like features. This comparison indicates that the decisive solubility-switching process is largely completed at the critical dose, whereas higher doses mainly improve morphological uniformity and process margin. The 3D surface morphologies obtained at other exposure doses are also provided (Figure S5). To evaluate the preliminary compatibility response of the patterned Hf-DEG film with fluorocarbon plasma exposure, the surface morphology was compared before and after CF_4_/Ar plasma treatment (Figure 4c,d and Figure S6). Before etching, the pattern exhibited a distinct step profile, although some residual roughness remained in the cleared region. After plasma exposure, the height contrast remained clear, the edge transition became sharper, and the cleared region appeared flatter, while the retained region maintained continuous topographic coverage (Figure S7). Quantitative roughness analysis further elucidated the effect of CF_4_/Ar plasma etching on the Hf-DEG pattern surface. The retained resist surface exhibited an Ra/Rq of 3.65/4.24 nm before etching and 1.58/1.95 nm after etching, while the cleared region showed an Ra/Rq of 4.32/7.69 nm before etching and 1.31/1.65 nm after etching (Table S4). These values, acquired from selected flat regions, confirm that the plasma-treated pattern retains a defined topographic contrast while the exposed HfO*_x_*-rich region remains mechanically robust under fluorocarbon plasma. Together, these results indicate that the Hf-DEG patterns formed at and above the critical dose remain well defined after fluorocarbon plasma treatment, suggesting compatibility with subsequent plasma-etching processes. While this test serves as a preliminary assessment rather than a comprehensive etch-rate or pattern-transfer validation, it is noteworthy that prolonged ambient storage of as-deposited Hf-DEG films alters the film chemistry (Figures S8 and S9), underscoring the need to control storage conditions and processing delay for practical implementation.

These observations extend the significance of the Hf-DEG resist beyond development contrast by showing that exposure converts the film into a more robust state for subsequent plasma etching. FTIR and XPS indicate that the exposed regions undergo loss of DEG-related organic coordination motifs and growth of more oxide-like Hf-O bonding, consistent with the formation of an HfO*_x_*-rich framework. This structural transformation provides a plausible basis for the preserved step profile and maintained topographic continuity after CF_4_/Ar plasma etching. Although a full quantitative etch analysis is beyond the scope of the present work, the retained pattern geometry after plasma exposure indicates that the state reached at the critical dose is sufficient not only for development but also for subsequent pattern transfer. Overall, the combined XPS, AFM, and plasma-etching results support the practical robustness of the Hf-DEG patterns after both development and plasma etching.

## 4. Conclusions

This work demonstrates an MLD-grown Hf-DEG dry photoresist that can be developed in 0.1 M HCl while exhibiting a critical dose of ~250 μC·cm^−2^ and reproducible negative-tone behavior with a continuous pattern down to 50 nm. By exploiting the molecular-level control inherent to MLD, the incorporation of DEG modifies the local coordination environment of the as-deposited hybrid film and leaves the unexposed regions with a larger fraction of acid-labile Hf-O-C motifs. As a result, the unexposed film is proposed to remain more susceptible to proton-assisted bond disruption and ligand exchange, which facilitates its removal under mild acidic conditions. Upon exposure, the observed attenuation of linker-related signals and the increased oxide-like Hf-O contribution suggest that the exposed regions evolve toward a more chemically resistant and less soluble HfO*_x_*-like framework. The ability of this system to operate in 0.1 M HCl therefore arises not simply from the oxide-rich character of the exposed film, which is common to many irradiated metal-containing hybrid resists, but from the combination of enhanced acid responsiveness in the unexposed regions and exposure-induced stabilization in the exposed regions. This DEG-engineered solubility contrast enables selective development under substantially milder chemical conditions than those commonly required for metal-containing resists while preserving practically relevant lithographic performance. Overall, these results show that controlling linker chemistry and the initial hybrid coordination environment is an effective route to improving developer compatibility in MLD-based metal-containing dry resists.

## Figures and Tables

**Table 1:** Comparison of development conditions and lithographic performance of representative Hf-based MLD hybrid resists.

Hf-Based System	Linker	Concentration of Developer	Tone	D_(c)_	Minimum Resolved Feature	Reference
Hf-EG	Ethylene glycol	3 M HCl	Negative	400 μC·cm^−2^	50 nm	[33]
Hf-DEG	Diethylene glycol	0.1 M HCl	Negative	250 μC·cm^−2^	50 nm	This work

D_(c)_ denotes the critical electron-beam dose required to produce a clear insoluble/retained-film response after development. The minimum resolved feature refers to the smallest continuous line pattern reported under the corresponding development conditions.

## Data Availability

The data that support the findings of this study are available from the corresponding author upon reasonable request.

## Supplementary Materials

The following supporting information can be downloaded at: https://www.mdpi.com/article/10.3390/nano16120726/s1, Table S1: Atomic percentage of Hf-DEG film before and after exposure; Table S2: XPS O 1s peak fit results before and after e-beam exposure; Table S3: XPS C 1s peak fit results before and after e-beam exposure; Table S4: AFM-derived step height and surface roughness parameters of Hf-DEG patterns before and after CF4/Ar plasma etching; Figure S1: Comparison of the dissolution performance of Hf-DEG films in HCl and KOH solutions; Figure S2: Images of the Hf-DEG dry photoresist after dose-matrix exposure and development: (a) optical microscope image; (b) camera image; Figure S3: SEM images of representative line patterns with trench widths of 20 nm obtained after exposure at 1500 μC·cm^−2^ and development in 0.1 M HCl; Figure S4: Line patterns obtained after EBL exposure at different doses and development in 0.1 M HCl. (a–g) show the patterns at doses of 100, 300, 500, 700, 900, 1100, and 1300, respectively; Figure S5: 3D topography after EBL exposure at different doses followed by development in 0.1 M HCl. (a–j) correspond to exposure doses of 50, 100, 150, 200, 300, 400, 450, 500, 1000, and 1500 μC·cm^−2^, respectively; Figure S6: Photograph of the sample after plasma etching; Figure S7: Comparison of step height before and after plasma etching. (a) before plasma etching, (b) after plasma etching; Figure S8: XPS results of the Hf-DEG film after 144 h storage in air. (a) C 1s, (b) O 1s; Figure S9: FTIR spectrum of Hf-DEG after 144 h storage in air.

## References

[1] Xia Y., Whitesides G.M. (1998). Soft Lithography. Annu. Rev. Mater. Sci..

[2] Ribeiro E., Violas A., Lopes T., Fernandes J., Teixeira J., Fernandes P., Salomé P. (2026). A Review of Nanoimprint Lithography for Improving the Optical Performance of Solar Cells. Microelectron. Eng..

[3] Gentili D., Valle F., Albonetti C., Liscio F., Cavallini M. (2014). Self-Organization of Functional Materials in Confinement. Acc. Chem. Res..

[4] Wang X., Tasdemir Z., Mochi I., Vockenhuber M., Van Lent-Protasova L., Meeuwissen M., Custers R., Rispens G., Hoefnagels R., Ekinci Y., Goldberg K.A. (2019). Progress in EUV Resists towards High-NA EUV Lithography. Proceedings of the Extreme Ultraviolet (EUV) Lithography X.

[5] Tao P., Sheng L., Wang Q., Cui H., Wang X., He X., Xu H. (2020). Photoresist for Extreme Ultraviolet Lithography. Proceedings of the 2020 International Workshop on Advanced Patterning Solutions (IWAPS).

[6] Radamson H.H., He X., Zhang Q., Liu J., Cui H., Xiang J., Kong Z., Xiong W., Li J., Gao J. (2019). Miniaturization of CMOS. Micromachines.

[7] Okoroanyanwu U. (2024). Lithographic Resists as Amazing Compact Imaging Systems—A Review. Micro Nano Eng..

[8] Kostko O., Xu B., Ahmed M., Slaughter D.S., Ogletree D.F., Closser K.D., Prendergast D.G., Naulleau P., Olynick D.L., Ashby P.D. (2018). Fundamental Understanding of Chemical Processes in Extreme Ultraviolet Resist Materials. J. Chem. Phys..

[9] Cen J., Deng Z., Liu S. (2024). Emerging Trends in the Chemistry of Polymeric Resists for Extreme Ultraviolet Lithography. Polym. Chem..

[10] Hasan M.W., Deeb L., Kumaniaev S., Wei C., Wang K. (2024). Recent Advances in Metal-Oxide-Based Photoresists for EUV Lithography. Micromachines.

[11] Ito H. (2003). Chemical Amplification Resists: Inception, Implementation in Device Manufacture, and New Developments. J. Polym. Sci. A Polym. Chem..

[12] Torok J., Re R.D., Herbol H., Das S., Bocharova I., Paolucci A., Ocola L.E., Ventrice C., Lifshin E., Denbeaux G. (2013). Secondary Electrons in EUV Lithography. J. Photopolym. Sci. Technol..

[13] Biafore J.J., Levinson H.J., Bailey T., Chunder A., Latypov A., Felix N.M., Goldberg K.A. (2018). Systematic Assessment of the Contributors of Line Edge Roughness in EUV Lithography Using Simulations. Proceedings of the Extreme Ultraviolet (EUV) Lithography IX.

[14] Li H., Peng R., Liu Z., Lian P., Chen J., Yu T., Zeng Y., Wang S., Guo X., Hu R. (2026). Recent Progress of Nonchemically-Amplified Resists for High-Resolution Lithography. Chin. J. Chem..

[15] Wang Y., Yu H., Wang L., Zhang Y., Zhu Z., Zhang Y., Lu Y., Ouyang C. (2025). Advances in Metal-Based Photoresist Materials for EUV Lithography and Lithographic Mechanisms. J. Mater. Chem. A.

[16] Kang Y.K., Lee S.J., Eom S., Kim B.G., Hwang C.-C., Kim M.-G. (2024). Recent Progress of Inorganic Photoresists for Next-Generation EUV Lithography. J. Mater. Chem. C.

[17] Wang D., Yi X., Zhang L. (2022). Non-Alkyl Tin-Oxo Clusters as New-Type Patterning Materials for Nanolithography. Sci. China Chem..

[18] Tseng Y.-F., Liao P.-C., Chen P.-H., Gau T.-S., Lin B.-J., Chiu P.-W., Liu J.-H. (2024). Highly Hydroxylated Hafnium Clusters Are Accessible to High Resolution EUV Photoresists under Small Energy Doses. Nanoscale Adv..

[19] Thakur N., Giuliani A., Nahon L., Castellanos S. (2020). Photon-Induced Fragmentation of Zinc-Based Oxoclusters for EUV Lithography Applications. J. Photopolym. Sci. Technol..

[20] Gardiner A.B., Burns S., Qin A., Willson C.G. (2001). Determination of Residual Casting Solvent Concentration Gradients in Resist Films by a “Halt Development” Technique. J. Vac. Sci. Technol. B Microelectron. Nanometer Struct. Process. Meas. Phenom..

[21] Mack C.A., Mueller K.E., Gardiner A.B., Sagan J.P., Dammel R.R., Willson C.G. (1998). Modeling Solvent Diffusion in Photoresist. J. Vac. Sci. Technol. B Microelectron. Nanometer Struct. Process. Meas. Phenom..

[22] Reynolds G.W., Taylor J.W. (1999). Factors Contributing to Sidewall Roughness in a Positive-Tone, Chemically Amplified Resist Exposed by x-Ray Lithography. J. Vac. Sci. Technol. B Microelectron. Nanometer Struct. Process. Meas. Phenom..

[23] Houle F.A., Hinsberg W.D., Sanchez M.I. (2002). Kinetic Model for Positive Tone Resist Dissolution and Roughening. Macromolecules.

[24] Hunek B., Cussler E.L. (2002). Mechanisms of Photoresist Dissolution. AIChE J..

[25] Meng X. (2017). An Overview of Molecular Layer Deposition for Organic and Organic–Inorganic Hybrid Materials: Mechanisms, Growth Characteristics, and Promising Applications. J. Mater. Chem. A.

[26] Sundberg P., Karppinen M. (2014). Organic and Inorganic–Organic Thin Film Structures by Molecular Layer Deposition: A Review. Beilstein J. Nanotechnol..

[27] Lee B.H., Yoon B., Abdulagatov A.I., Hall R.A., George S.M. (2013). Growth and Properties of Hybrid Organic-inorganic Metalcone Films Using Molecular Layer Deposition Techniques. Adv. Funct. Mater..

[28] George S.M. (2010). Atomic Layer Deposition: An Overview. Chem. Rev..

[29] Wang X., Guo T., Shan Y., Zhang O., Dong H., Liu J., Luo F. (2024). An Aluminum-Based Hybrid Film Photoresist for Advanced Lithography by Molecular Layer Deposition. J. Mater. Chem. C.

[30] Shan Y., Wang X., Zheng X., Zhao X., Feng Z., Wang W., Cheng Y., Liu H., Tan K., Luo F. (2025). Study of Molecular Layer Deposition of Zinc-Based Hybrid Film as Photoresist. Appl. Surf. Sci..

[31] Lewis J., Than L.V., Bent S.F. (2026). Aromatic Organic-Tin Hybrid Films as Electron Beam Resists. Appl. Surf. Sci..

[32] Kim D.G., Ha K., Kim H., Kim W.-H., Park T.J., Ahn J.-H. (2025). Molecular Layer Deposition of Tin-Based Organic–Inorganic Hybrid Films as Photoresists. Appl. Surf. Sci..

[33] Shi J.W., Ravi A., Richey N.E., Gong H.X., Bent S.F. (2022). Molecular Layer Deposition of a Hafnium-Based Hybrid Thin Film as an Electron Beam Resist. ACS Appl. Mater. Interfaces.

[34] Jung J., Park R., Jeon N. (2026). Vacuum-Processed Hybrid Resists for Advanced Lithography: Molecular Layer Deposition and Sequential Infiltration Synthesis. Nanoscale.

[35] Than L.V., D’Acunto G., Harake M., Im H., Kostko O., Bent S.F. (2025). Effect of Networking Density on the Patterning Performance of Molecular Layer Deposited Alucone Electron Beam/EUV Resists. ACS Appl. Mater. Interfaces.

[36] Qiao Y., Shi G., Zhang O., Li Y., Vockenhuber M., Ekinci Y., Luo F., Zhang L. (2024). Heterometallic Ti-Zr Oxo Nanocluster Photoresists for Advanced Lithography. Sci. China Mater..

[37] Chen H., Huang X., Zhao Y., Zhao J., Chen P., Peng X. (2024). Balancing Sensitivity and Resolution by Feedback Regulation of Free Radicals from Sn-C Bonds in Tin-Oxygen Clusters EBL Photoresist. Sci. China Mater..

[38] Chen H., Peng Y., Fu H., Han F., Shi G., Luo F., Zhao J., Zhou D., Chen P., Peng X. (2024). Effect of Free Radicals on Irradiation Chemistry of a Double-Coordination Organotin (Sn_4_) Photoresist by Adjusting Alkyl Ligands. CCS Chem..

[39] Liu J., Kang W. (2023). New Chemically Amplified Positive Photoresist with Phenolic Resin Modified by GMA and BOC Protection. Polymers.

[40] Jung H., Jang D., Jung J., Lee C., Jang A. (2024). Application of an Ozone-Activated Peroxymonosulfate Process to Effectively Degrade Tetramethylammonium Hydroxide (TMAH) in Semiconductor Wastewater. J. Water Process Eng..

[41] Wang Y., Zhang Z., Jiang C., Xu T. (2013). Electrodialysis Process for the Recycling and Concentrating of Tetramethylammonium Hydroxide (TMAH) from Photoresist Developer Wastewater. Ind. Eng. Chem. Res..

[42] Jiang J., Jung B., Thompson M.O., Ober C.K. (2019). Chemical Reaction and Diffusion Kinetics during Laser-Induced Submillisecond Heating for Lithographic Applications. J. Vac. Sci. Technol. B Nanotechnol. Microelectron. Mater. Process. Meas. Phenom..

[43] Lee J., Lee S., Choi Y., Lee S. (2023). Treatment of Semiconductor Wastewater Containing Tetramethylammonium Hydroxide (TMAH) Using Nanofiltration, Reverse Osmosis, and Membrane Capacitive Deionization. Membranes.

[44] Innocenzi V., Zueva S.B., Ippolito N.M., Ferella F., Prisciandaro M., Vegliò F. (2022). A Review of the Existing and Emerging Technologies for Wastewaters Containing Tetramethyl Ammonium Hydroxide (TMAH) and Waste Management Systems in Micro-Chip Microelectronic Industries. Chemosphere.

[45] Fallica R., Chen S., De Simone D., Suh H.S. (2022). Adhesion and Collapse of Extreme Ultraviolet Photoresists and the Role of Underlayers. J. Micro/Nanopatterning Mater. Metrol..

[46] Gronheid R. (2010). Impact of Development Chemistry on Extreme Ultraviolet Resist Performance. J. Vac. Sci. Technol. B Nanotechnol. Microelectron. Mater. Process. Meas. Phenom..

[47] Harumoto M., Dos Santos A.F., Santillan J.J., Itani T., Kozawa T. (2023). Stochastic Defect Generation Depending on Tetraalkylhydroxide Aqueous Developers in Extreme Ultraviolet Lithography. Jpn. J. Appl. Phys..

[48] Santillan J.J., Shimizu K., Otogawa R., Itani T. (2022). Effect of Alternative Developer Solutions on EUVL Patterning. J. Photopolym. Sci. Technol..

[49] Rao A., Kang S., Vogt B.D., Prabhu V.M., Lin E.K., Wu W.-L., Muthukumar M. (2006). Effect of Deprotection Extent on Swelling and Dissolution Regimes of Thin Polymer Films. Langmuir.

[50] Moore J.C., Fedynyshyn T.H. (2002). Successful Photoresist Removal: Incorporating Chemistry, Conditions, and Equipment. Advances in Resist Technology and Processing XIX.

[51] Prabhu V.M., Wang M.X., Jablonski E.L., Vogt B.D., Lin E.K., Wu W.-L., Goldfarb D.L., Angelopoulos M., Ito H., Sturtevant J.L. (2004). Fundamentals of Developer-Resist Interactions for Line-Edge Roughness and Critical Dimension Control in Model 248-Nm and 157-Nm Photoresists. Advances in Resist Technology and Processing XXI.

[52] Hussain M.M., Moumen N., Zhang Z., Womack B.F. (2006). Metal Wet Etch Issues and Effects in Dual Metal Gate Stack Integration. J. Electrochem. Soc..

[53] Cui H., Luo J., Xu J., Gao J., Xiang J., Tang Z., Wang X., Lu Y., He X., Li T. (2015). Investigation of TaN as the Wet Etch Stop Layer for HKMG-Last Integration in the 22 Nm and beyond Nodes CMOS Technology. Vacuum.

[54] Choi S.-H., Kreider M.E., Nielander A.C., Stevens M.B., Kamat G., Koo J.E., Bae K.H., Kim H., Yoon I.Y., Yoon B.U. (2022). Origins of Wear-Induced Tungsten Corrosion Defects in Semiconductor Manufacturing during Tungsten Chemical Mechanical Polishing. Appl. Surf. Sci..

[55] Cooper E.I., Rajaram R., Payne M., Lippy S. (2012). Selective High-Throughput TiN Etching Methods. Solid State Phenom..

[56] Li Y., Xu Q. (2009). TaN Wet Etch for Application in Dual-Metal-Gate Integration Technology. J. Semicond..

[57] Chyou S.D., Shih H.C., Chen T.T. (1993). On the Corrosion Characterization of Titanium Nitride in Sulfuric Acid Solution. Corros. Sci..

[58] Broussous L., Puyrenier W., Rebiscoul D., Rouessac V., Ayral A. (2009). Porous Low-k Wet Etch in HF-Based Solutions: Focus on Cleaning Process Window, “Pore-Sealing” and “k Recovery”. Solid State Phenom..

[59] Broussous L., Puyrenier W., Rebiscoul D., Rouessac V., Ayral A. (2007). Porosity and Structure Evolution of a SiOCH Low k Material during Post-Etch Cleaning Process. Microelectron. Eng..

[60] Zhao Y., He B., Yang J., Liu Y., Zhang T., Wang F. (2025). Critical Role of Intermetallic Particles in the Corrosion of 6061 Aluminum Alloy and Anodized Aluminum Used in Semiconductor Processing Equipment. Acta Metall. Sin. (Engl. Lett.).

[61] Ren J., Xu L., Luo J., Li Z., Li B., Shi X., Xu L., Thi Bang L., Fu Q. (2023). Hydrothermal Oxidation of Titanium Nitride Coating for Enhanced Corrosion Resistance in Fluoride-Containing Acidic Solution. Mater. Lett..

[62] Lin C.-C., Yang C.-C., Ger J., Deng J.-F., Hung D.-Z. (2010). Tetramethylammonium Hydroxide Poisoning. Clin. Toxicol..

[63] Dameron A.A., Seghete D., Burton B.B., Davidson S.D., Cavanagh A.S., Bertrand J.A., George S.M. (2008). Molecular Layer Deposition of Alucone Polymer Films Using Trimethylaluminum and Ethylene Glycol. Chem. Mater..

[64] Seghete D., Hall R.A., Yoon B., George S.M. (2010). Importance of Trimethylaluminum Diffusion in Three-Step ABC Molecular Layer Deposition Using Trimethylaluminum, Ethanolamine, and Maleic Anhydride. Langmuir.

[65] Jain H., Poodt P. (2021). About the Importance of Purge Time in Molecular Layer Deposition of Alucone Films. Dalton Trans..

[66] Philip A., Mai L., Ghiyasi R., Devi A., Karppinen M. (2022). Low-Temperature ALD/MLD Growth of Alucone and Zincone Thin Films from Non-Pyrophoric Precursors. Dalton Trans..

[67] Ravi A., Than L.V., Lewis J., Shi J., Werbrouck A., Han J., Mattinen M., Bent S.F. (2024). Comparison of Al- and Hf-Based Hybrid Photoresists Grown by Molecular Layer Deposition for Extreme Ultraviolet Lithography. J. Vac. Sci. Technol. A.

[68] Oleksak R.P., Ruther R.E., Luo F., Amador J.M., Decker S.R., Jackson M.N., Motley J.R., Stowers J.K., Johnson D.W., Garfunkel E.L. (2018). Evaluation of Thermal and Radiation Induced Chemistries of Metal Oxo–Hydroxo Clusters for next-Generation Nanoscale Inorganic Resists. ACS Appl. Nano Mater..

[69] Mattson E.C., Cabrera Y., Rupich S.M., Wang Y., Oyekan K.A., Mustard T.J., Halls M.D., Bechtel H.A., Martin M.C., Chabal Y.J. (2018). Chemical Modification Mechanisms in Hybrid Hafnium Oxo-Methacrylate Nanocluster Photoresists for Extreme Ultraviolet Patterning. Chem. Mater..

[70] Vemuri V., King S.W., Thorpe R., Jones A.H., Gaskins J.T., Hopkins P.E., Strandwitz N.C. (2024). Chemical, Structural, and Electrical Changes in Molecular Layer-Deposited Hafnicone Thin Films after Thermal Processing. ACS Appl. Electron. Mater..

[71] Le D.N., Veyan J.-F., Chu T.T.H., Wilson L.A., Pham L., Sung H.D., Lee W.-I., Tiwale N., Lee J., Choi R. (2025). Electron-Induced Chemical Transformation of Vapor-Phase Synthesized Hybrid Resist Materials for EUV and beyond EUV Lithography. MRS Adv..

[72] Gengenbach T.R., Major G.H., Linford M.R., Easton C.D. (2021). Practical Guides for X-Ray Photoelectron Spectroscopy (XPS): Interpreting the Carbon 1s Spectrum. J. Vac. Sci. Technol. A Vac. Surf. Film..

[73] Shard A.G. (2020). Practical Guides for X-Ray Photoelectron Spectroscopy: Quantitative XPS. J. Vac. Sci. Technol. A.

[74] Luo X., Li Y., Yang H., Liang Y., He K., Sun W., Lin H.-H., Yao S., Lu X., Wan L. (2018). Investigation of HfO_2_ Thin Films on Si by X-Ray Photoelectron Spectroscopy, Rutherford Backscattering, Grazing Incidence X-Ray Diffraction and Variable Angle Spectroscopic Ellipsometry. Crystals.

[75] Raeliarijaona A., Cohen R.E. (2022). First-Principles Calculations of Raman and Infrared Spectroscopy for Phase Identification and Strain Calibration of Hafnia. Appl. Phys. Lett..

[76] Cui X., Tuokedaerhan K., Cai H., Lu Z. (2022). Effect of Annealing Temperature on the Microstructure and Optical Properties of Lanthanum-Doped Hafnium Oxide. Coatings.

[77] Alam F., He G., Yan J., Wang W. (2023). All-Water-Driven High-k HfO_2_ Gate Dielectrics and Applications in Thin Film Transistors. Nanomaterials.

[78] Wang X., Xu Y., Zhang O., Dong H., Sun X., Luo F. (2026). Positive and Negative Dual-Type Hafnium-Based Hybrid Dry Photoresist for Nanolithography. ACS Appl. Mater. Interfaces.

