# Supplementary material for: An Ether-Containing Hafnium–Diethylene Glycol Dry Resist Prepared by Molecular Layer Deposition for Mild-Acid Development
Source: Nanomaterials (Basel). 2026 Jun 11;16(12):726. doi: 10.3390/nano16120726 (PMC13304698; doi:10.3390/nano16120726)
Supplement: Supplementary file 1 [file nanomaterials-16-00726-s001.zip › nanomaterials-4323882-supplementary.pdf]

nanomaterials

Supporting Information

An Ether-Containing Hafnium–Diethylene Glycol Dry Resist Prepared by Molecular Layer Deposition for Mild-Acid Development

Chao Shi[1,2], Yixian Wang[1,2], Zimai Wang[1,2], Yumo Tian[1,2], Kuanlin Chen[1,2], Linyang Li[1,2], Xianhaoyan Chen[1,2], Yuan Cai[3] and Tuo Wang[1,2,4,5,6,*]

[1] School of Chemical Engineering & Technology, Key Laboratory for Green Chemical Technology of Ministry of Education, Collaborative Innovation Center for Chemical Science & Engineering, Tianjin University, Tianjin 300072, China
[2] International Joint Laboratory of Low-carbon Chemical Engineering of Ministry of Education, Tianjin 300350, China
[3] Zhejiang Institute of Tianjin University, Ningbo, Zhejiang 315201, China
[4] Haihe Laboratory of Sustainable Chemical Transformations, Tianjin 300192, China
[5] Joint School of National University of Singapore and Tianjin University, International Campus of Tianjin University, Fuzhou 350207, China
[6] National Industry-Education Platform of Energy Storage, Tianjin 300350, China
* Correspondence: wangtuo@tju.edu.cn

Academic Editor: Firstname Lastname

Received: date
Revised: date
Accepted: date
Published: date

Copyright: © 2026 by the authors. Submitted for possible open access publication under the terms and conditions of the Creative Commons Attribution (CC BY) license.

Table S1 Atomic percentage of Hf-DEG film before and after exposure

	Hf-DEG film Atomic concentration (%)	Exposed Hf-DEG film Atomic concentration (%)
Hf	26.82	28.2
O	47.53	43.59
C	25.65	28.21

Table S2 XPS O 1s peak fit results before and after e-beam exposure

	Element/ Transition	Peak Energy (eV)	Concentration (at.%)
Unexposed sample	O 1s	531.7	100
Exposed sample	O 1s	530.3	22.0
	O 1s	531.8	78.0

Table S3 XPS C 1s peak fit results before and after e-beam exposure

	Element/ Transition	Peak Energy (eV)	Concentration (at.%)
Unexposed sample	C 1s	284.8	59.6
	C 1s	286.4	37.1
	C 1s	289.0	3.3
Exposed sample	C 1s	284.8	79.0
	C 1s	286.3	17.6
	C 1s	289.0	3.4

Table S4 AFM-derived surface roughness parameters of Hf-DEG patterns before and after CF_4/Ar plasma etching

	Retained resist surface		Cleared region	
	Before etching	After etching	Before etching	After etching
Ra (nm)	3.56	1.58	4.32	1.31
Rq (nm)	4.24	1.95	7.69	1.65

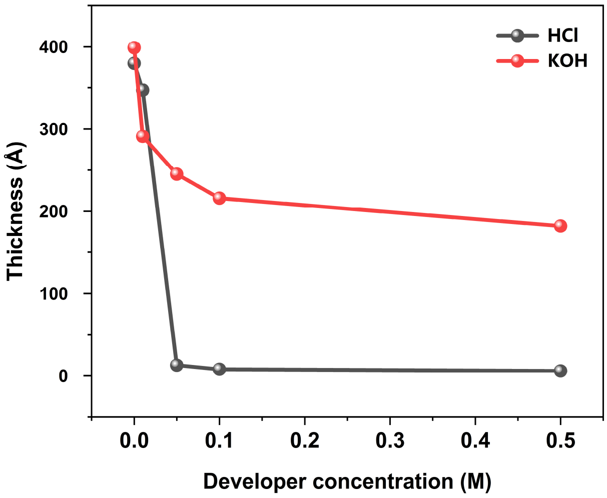

Figure S1. Comparison of the dissolution performance of Hf-DEG films in HCl and KOH solutions.

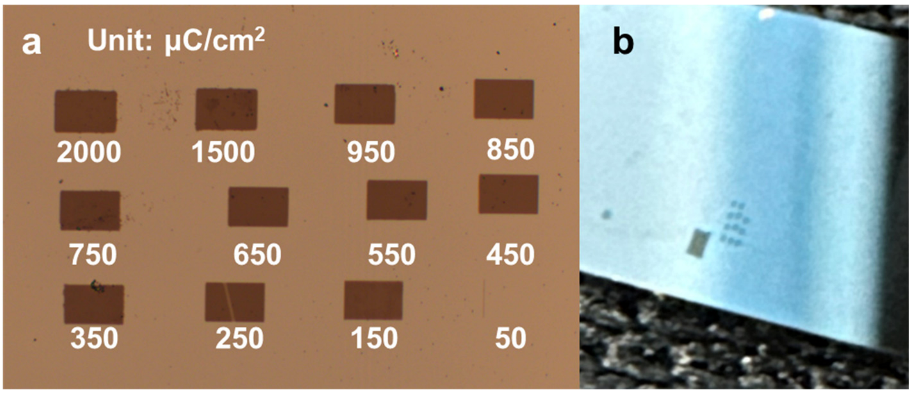

Figure S2. Images of the Hf-DEG dry photoresist after dose-matrix exposure and development: (a) optical microscope image; (b) camera image.

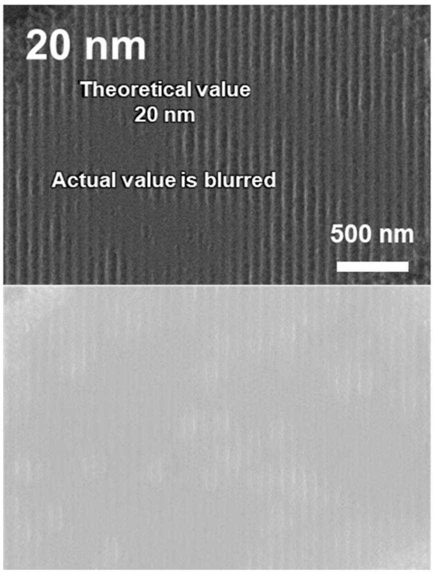

Figure S3. SEM images of representative line patterns with trench widths of 20 nm obtained after exposure at 1500 μC·cm^{-2} and development in 0.1 M HCl.

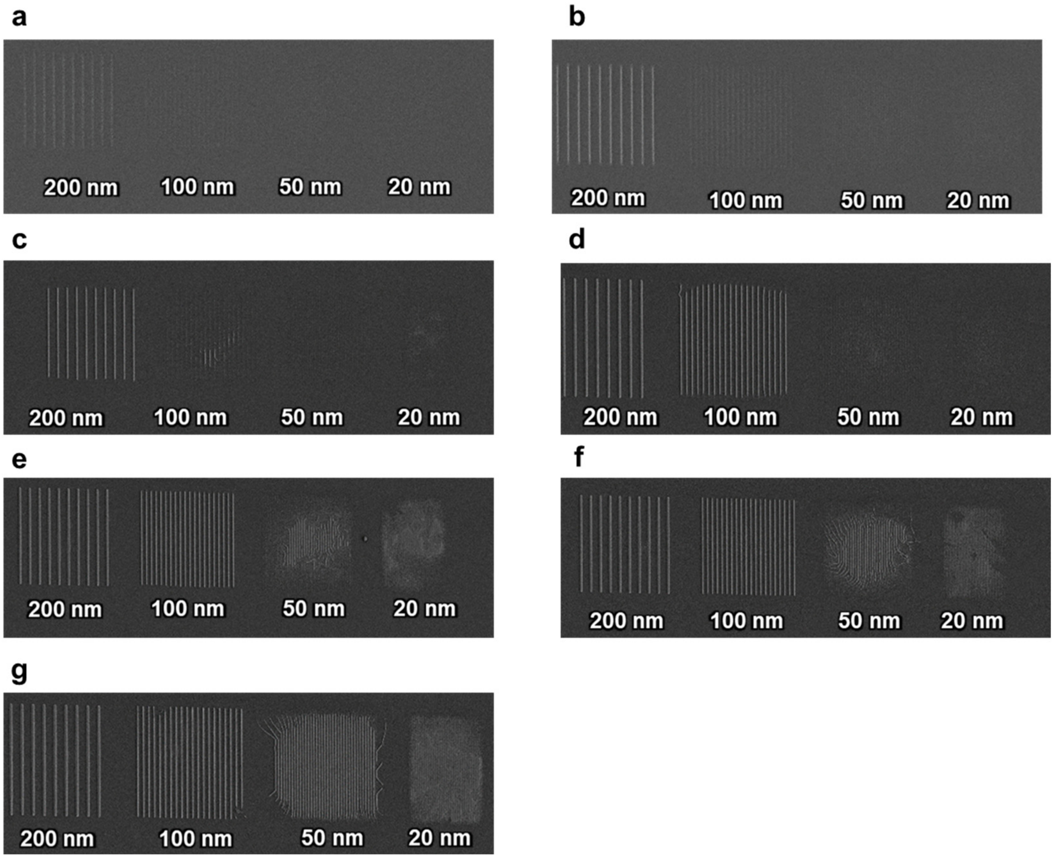

Figure S4. Line patterns obtained after EBL exposure at different doses and development in 0.1 M HCl. (a-g) show the patterns at doses of 100, 300, 500, 700, 900, 1100, and 1300 μC·cm^{-2}, respectively.

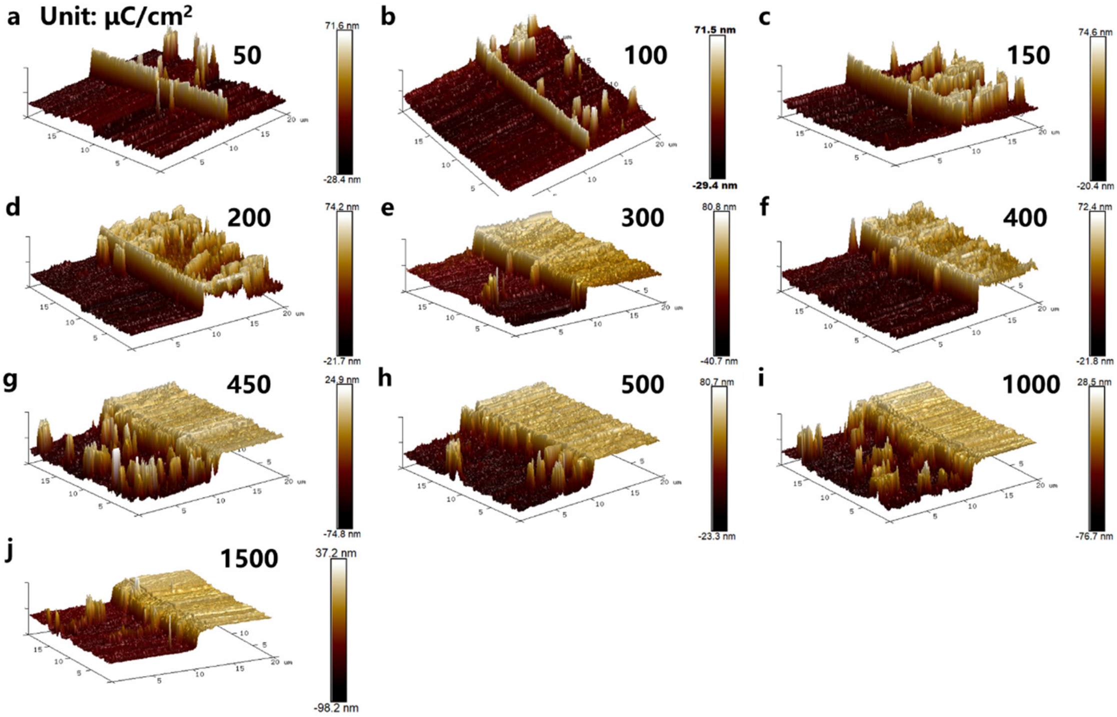

Figure S5. 3D topography after EBL exposure at different doses followed by development in 0.1 M HCl. (a-j) correspond to exposure doses of 50, 100, 150, 200, 300, 400, 450, 500, 1000, and 1500 μC·cm^{-2}, respectively.

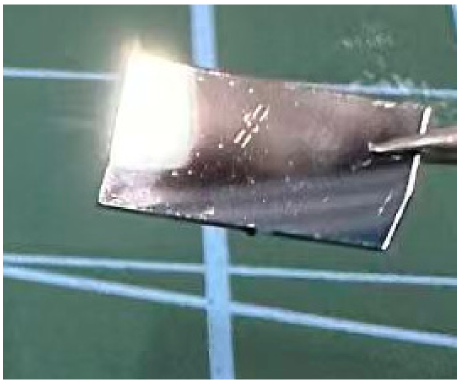

Figure S6. Photograph of the sample after plasma etching.

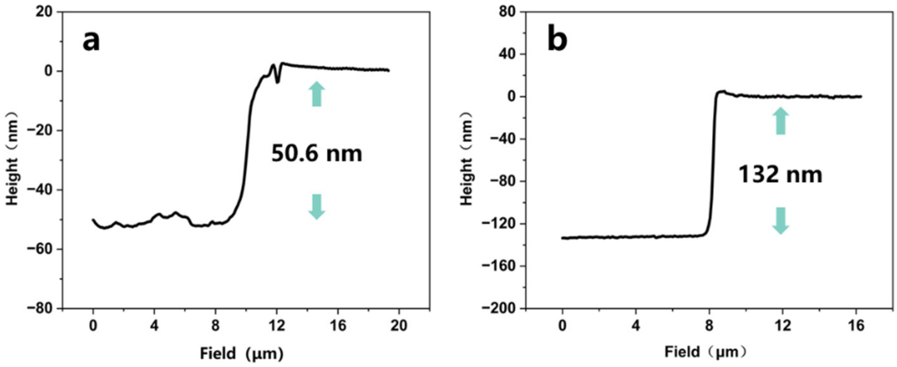

Figure S7. Comparison of step height before and after plasma etching. (a) before plasma etching, (b) after plasma etching.

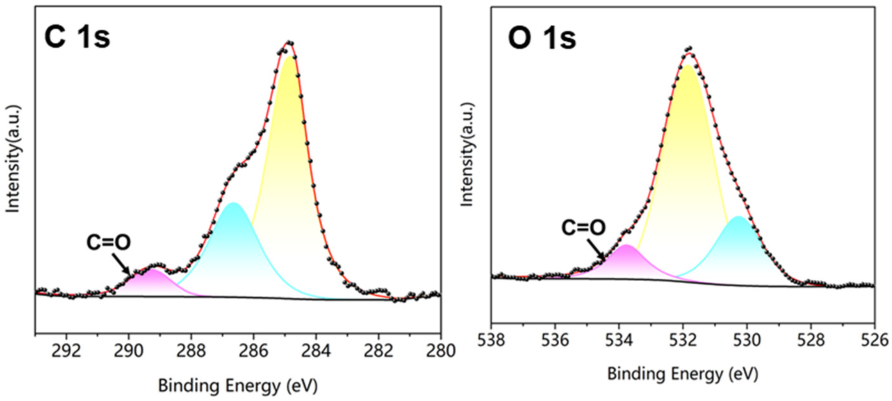

Figure S8. XPS results of the Hf-DEG film after 144 h storage in air. (a) C 1s, (b) O 1s.

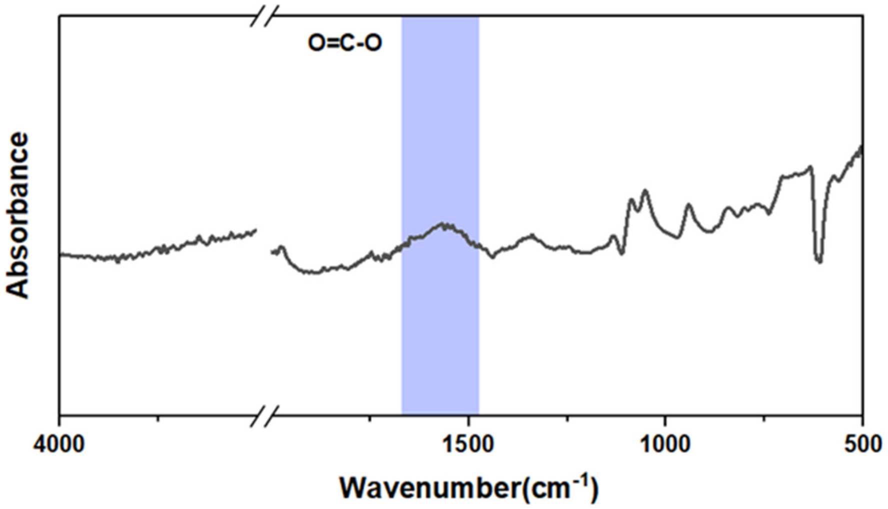

Figure S9. FTIR spectrum of Hf-DEG after 144 h storage in air.
